# Breastfeeding at the workplace: a systematic review of interventions to improve workplace environments to facilitate breastfeeding among working women

**DOI:** 10.1186/s12939-021-01432-3

**Published:** 2021-04-29

**Authors:** Mireya Vilar-Compte, Sonia Hernández-Cordero, Mónica Ancira-Moreno, Soraya Burrola-Méndez, Isabel Ferre-Eguiluz, Isabel Omaña, Cecilia Pérez Navarro

**Affiliations:** 1 Research Center for Equitable Development EQUIDE, Universidad Iberoamericana, Prolongación Paseo de la Reforma 880, Lomas de Santa Fe, 01219 Mexico City, Mexico; 2 Department of Health, Universidad Iberoamericana, Prolongación Paseo de la Reforma 880, Lomas de Santa Fe, 01219 Mexico City, Mexico

**Keywords:** Breastfeeding, Working mothers, Lactation/breastfeeding rooms, Breastmilk pumping, Breastfeeding education, Workplace interventions

## Abstract

**Background:**

Breastfeeding can be affected by maternal employment. This is important considering that in 2019, 47.1% of women globally participated in the labor force. The aim of this study was to review workplace interventions to promote, protect and support breastfeeding practices among working mothers globally.

**Methods:**

A systematic review was conducted following the guidance of the Preferred Reporting Items for Systematic Reviews and Meta-Analyses (PRISMA). Observational, experimental and qualitative peer-reviewed studies in English and Spanish, published between 2008 and 2019 were included. The review focused on working women who were pregnant, breastfeeding or who recently had a child, and women’s working environments. The outcomes of interest included breastfeeding intentions, initiation, exclusivity and duration, confidence in breastfeeding or breastmilk extraction, and perceived support at workplace. Quality was assessed according to National Institute for Health and Care Excellence (NICE) checklist for systematic reviews. It was registered on PROSPERO (#140624).

**Results:**

Data was extracted from 28 quantitative and 9 qualitative studies. The most common interventions were designated spaces for breastfeeding or breastmilk extraction (*n* = 24), and the support from co-workers (*n* = 20). The least common interventions were providing breast pumps (*n* = 4) and giving mothers the flexibility to work from home (*n* = 3). Studies explored how interventions affected different breastfeeding outcomes including breastfeeding duration, breastfeeding exclusivity, confidence in breastmilk expression, and breastfeeding support. The evidence suggests that workplace interventions help increase the duration of breastfeeding and prevent early introduction of breastmilk substitutes. Having a lactation space, breastmilk extraction breaks, and organizational policies are key strategies. However, to achieve equitable working conditions for breastfeeding mothers, organizational and interpersonal changes need to occur as well.

**Conclusions:**

The systematic review revealed that interventions at the workplace are important in protecting, promoting and supporting breastfeeding among working mothers. To achieve equitable work environments and fair nutritional opportunities for infants of working mothers, interventions should focus at the three ecological layers – individual, interpersonal, and organizational. The quality of studies can be improved. There is a need for studies assessing impacts of workplace interventions on infant feeding practices, mothers’ self-esteem and outcomes such productivity and abstentionism.

**Table:** 

This article is a part of the Interventions and policy approaches to promote equity in breastfeeding collection, guest-edited by Rafael Pérez-Escamilla, PhD and Mireya Vilar-Compte, PhD	

## Introduction

Breastfeeding is the best source of infant nutrition and contributes to maternal health. The World Health Organization (WHO) and the United Nations International Children’s Emergency Fund (UNICEF) recommend to exclusively breastfeed during the first six months of life and to continue breastfeeding with complementary foods at least until the age of 2 [1]. However, breastfeeding practices are still far from current recommendations, for example, globally the prevalence of exclusive breastfeeding among infants younger than 6 months is 37% [2]. Breastfeeding practices can be affected by several factors, employment among them [3]. Maternal employment without adequate support has been previously described as a barrier to breastfeeding [4–6]. Work-related issues have been identified as a major reason of why mothers do not initiate breastfeeding or wean their babies sooner [7]. Hence, public policies are needed for working mothers to effectively enforce their choice to optimally breastfeed. This is especially important considering that in 2019 47.1% of women globally participated in the labor force [8]. Without adequate policies, women in the labor force and their babies will keep facing inequities in terms of infant nutrition and employment choices, and the right of women to combine motherhood and professional development would be jeopardized.

Several strategies have been proposed to enhance breastfeeding among working women, such as early postpartum support, maternity leave policies, teleworking, flexible working hours and access to space and time to extract human milk [6, 9]. While there is an increasing body of literature about the association between maternity leave benefits and increased duration of optimal breastfeeding, less is known in terms of the impacts of policies at the workplace in promoting, supporting and protecting breastfeeding. These policies can have substantial effects in shaping breastfeeding of working mothers; examples of contextual elements that affect such choices are the space and time to extract milk, support from colleagues and supervisors, family arrangements and support to breastfeeding women while at the workplace, the existence of explicit policies to support breastfeeding working mothers by firms, amongst others. Hence, given the increasing share of women who are active in the labor market, the workplace is a fundamental setting to intervene to support women who decide to continue with breastfeeding once they return to work. Workplace breastfeeding interventions fulfill different social objectives such as infant nutrition, gender equality and economic development, all which can contribute to equitable social outcomes.

To gather a clearer understanding of the types of interventions at the workplace that can facilitate maintaining optimal breastfeeding practices once women return to work, the aim of the current study was to conduct a global systematic literature review exploring workplace interventions to promote, protect and support breastfeeding practices among working mothers.

## Methods

The protocol for this systematic review was registered in PROSPERO prior to starting the literature search (#140624). This systematic review followed the guidance of the Preferred Reporting Items for Systematic Reviews and Meta-Analyses (PRISMA) [10, 11].

### Inclusion and exclusion criteria

Observational, experimental and qualitative peer-reviewed studies in English and Spanish were included if they addressed interventions supporting breastfeeding in the workplace, including: written policies to support breastfeeding employees; breastfeeding education for employees and/or counselling for breastfeeding women at the workplace; designated private or semi-private spaces for breastfeeding or expressing milk; flexible scheduling to support milk expression during work (i.e. breastfeeding/expression breaks); giving mothers options to work from home (i.e., home-office) or reduced hours; and providing breast pumps at the workplace. In addition, we included studies describing support from co-workers and supervisors, a relevant factor in fostering efficacy among working women [12]. While paternal leave is recognized as a key intervention to foster breastfeeding it requires policy designs that often times are not per se a workplace policy but rather a social protection intervention [13]. Hence, paternal leave interventions were excluded. Similarly, on-site or near-site child care facilities were not considered, as they would require addressing literature that is not necessarily within the workplace space. However, we acknowledge that this is a fundamental complement to workplace breastfeeding policies.

The review focused on working women who were pregnant, breastfeeding or who recently had a child (i.e., 5 years) and women’s working environments, which included perceptions about breastfeeding or breastfeeding support among supervisors, managers and/or co-workers. From an analytical perspective, observational studies or in-depth cases informing the breastfeeding experience of working women, and comparative studies assessing differential impacts or associations between breastfeeding interventions in the workplace and breastfeeding outcomes were included. The outcomes of interest included breastfeeding intentions, initiation, exclusivity and duration, confidence in breastfeeding or breastmilk extraction and perceived support at the workplace. Table 1 summarizes the inclusion and exclusion criteria.

**Table 1:** Inclusion criteria for breastfeeding interventions in the workplace

Criteria	Inclusion
Type of Literature	Peer reviewed journal articles.
Type of Studies	Qualitative or quantitative empirical studies (observational or experimental).
Intervention	Breastfeeding interventions at the workplace: -written policies -breastfeeding education and/or counselling at the workplace -designated private or semi-private spaces -flexible scheduling -options to work from home or reduced hours - breast pumps at the workplace -co-workers and supervisors’ support
Level of Analysis	Analyses of working women (pregnant, breastfeeding or who had a child during the last 5 years) and their workplace context.
Analytical Perspective	Descriptive analyses or in-depth cases looking at the experiences of pregnant and recent mothers around their breastfeeding choices. Comparative analyses assessing interventions at the workplace affecting breastfeeding outcomes.
Outcome	Breastfeeding intentions; breastfeeding initiation, exclusivity and duration; breastfeeding self-efficacy and perceived support.
Target Population	Employed women who were pregnant, breastfeeding or who had a child during the last 5 years.

### Search strategy

Four bibliographical databases (PubMed, Web of Science, Scielo and Scopus) were systematically searched for studies published between January 2008 and June 2019. This time frame was selected considering that during the early 2000s several international organizations highlighted the need to protecting breastfeeding among the increasing share of working mothers. In 2000, the International Labor Organization (ILO) explicitly stated actions in the Maternity Protection Convention (#183). Similarly, in 2003 the WHO and UNICEF recommended “enacting imaginative legislation protecting the breastfeeding rights of working women” to be enforced by governments [14]. As it took a range of time for countries to start implementing actions considering such recommendations, a 5-year period was deemed necessary for the scientific literature to start reporting descriptions and evaluations. Relevant literature was identified following the search algorithms summarized in Table 2. Free-text terms were used to generate search strategies for each database. Studies identified through each database were imported to Excel and then, duplicates were identified and removed. The studies were subsequently imported to EndNote [15]. In 2017, Dinour and Szaro [16] conducted a literature review of employer-based programs. While such review did not include qualitative studies, excluded literature in languages different than English, and did not assess the quality of the papers, it served as a standard to compare the convergence of our search algorithm, which was adequate and captured the same studies within the common search years.

**Table 2:** Boolean search system

**Search system**	
“((Breast Feeding OR partial breastfeeding OR Predominant breastfeeding OR Feeding, Breast OR Breast Feeding, Exclusive OR Breastfeeding, Exclusive OR Exclusive Breastfeeding)) AND (Workplaces OR Work Location OR Location, Work OR Locations, Work OR Work Locations OR Work-Site OR Work Site OR Work-Sites OR Work Place OR Place, Work OR Places, Work OR Work Places OR Job Site OR Job Sites OR Site, Job OR Sites, Job OR Worksite OR Worksites))”	

### Study selection and quality assessment

In the first phase, abstracts were reviewed by six of the authors (MVC, SHC, MAM, IF, IO, MC). Two authors independently assessed the same abstract and then decisions were compared; whenever there was dissent, another of the authors (SBM) reviewed the abstract and decided. In the next phase, articles were retrieved and independently assessed for eligibility. Papers were assessed for quality according to National Institute for Health and Care Excellence (NICE) checklists for systematic reviews. Quantitative studies were assessed based on the following criteria: description of the setting and context; definition of eligibility and process of recruitment; validity of outcome measures; whether the outcome measure could objectively or subjectively capture the construct of interest; if the study presented coherent sample size and power estimations or justifications; the capacity of the study to measure effects or associations; whether estimations were adjusted for confounders and covariates; confidence intervals and *p*-values; and, finally, addressing sources of bias and external validity. A similar approach was used for qualitative studies, NICE has a specific checklist in which the following aspects are assessed: the research question, purpose and rationale of the study; the data collection process; the role of the researcher within the study; context bias and setting; triangulation; analytical strategy, saturation and coding process; presentation of the findings and its links to the purpose of the study; plausibility and coherence of the conclusions; as well as ethical considerations on how the study was conducted. Each study was graded with the corresponding checklist by two of the authors (MVC, SHC, MAM, IF, IO, MC), who were standardized beforehand. A third researcher (SBM) helped reaching consensus in divergent grades.

### Data extraction

For selected manuscripts, data was extracted through a predetermined format by six authors (MVC, SHC, MAM, IO, CP, SBM) who were previously harmonized. For quantitative studies, extracted data included country/city; specific population and/or setting; design; type of breastfeeding intervention; outcome variable; type of analysis, and size of the effects or associations. For qualitative studies, extracted data included country/city; specific population and/or setting; type of breastfeeding intervention; design; data collection; type of analysis; and key conclusions.

## Results

### Study characteristics

**Fig. 1:**
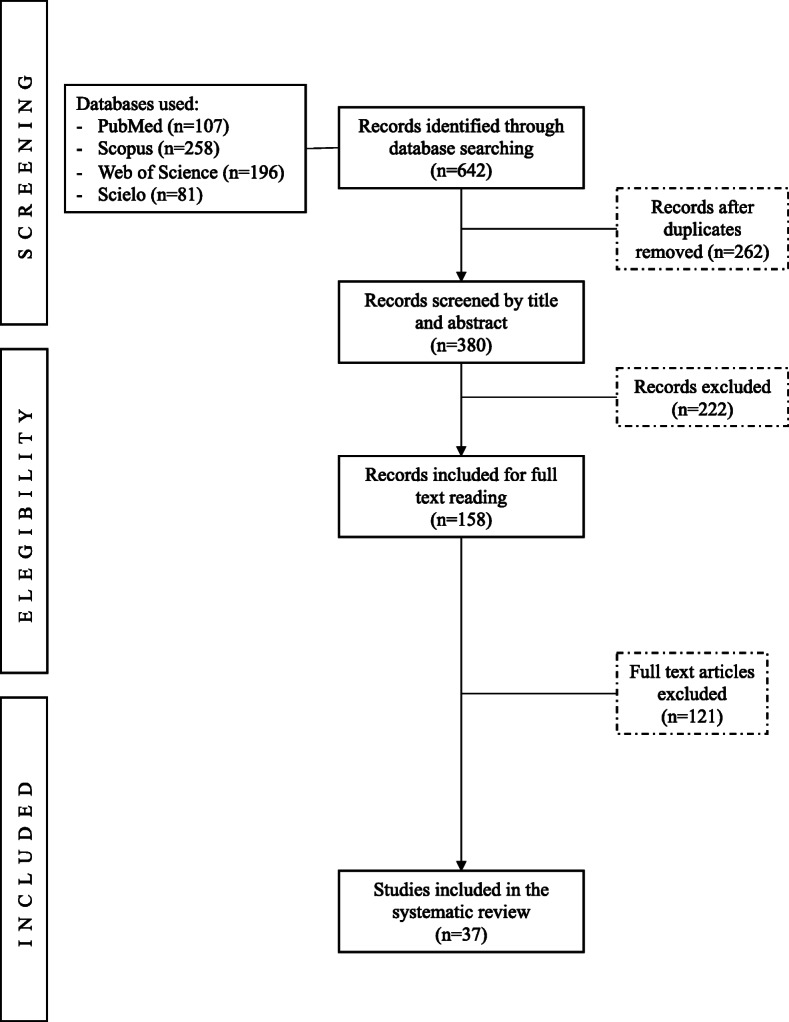
Preferred Reporting Items for Systematic Reviews and Meta-Analyses diagram

A summary of the search results is shown in Fig. 1. After duplicate studies were removed, the titles and abstracts from 380 records were screened for inclusion, of which 158 articles were fully reviewed to determine eligibility. Data was extracted from 28 quantitative and 9 qualitative studies were fully screened (*n* = 37). Among the quantitative studies, the majority were cross-sectional studies (*n* = 24) and the rest had a longitudinal or prospective cohort design (*n* = 4). The qualitative studies followed a phenomenological approach (*n* = 4), grounded theory (*n* = 1), investigation action methodology (*n* = 2), ethnography (*n* = 1) and online qualitative questionnaire (*n* = 1).

**Fig. 2:**
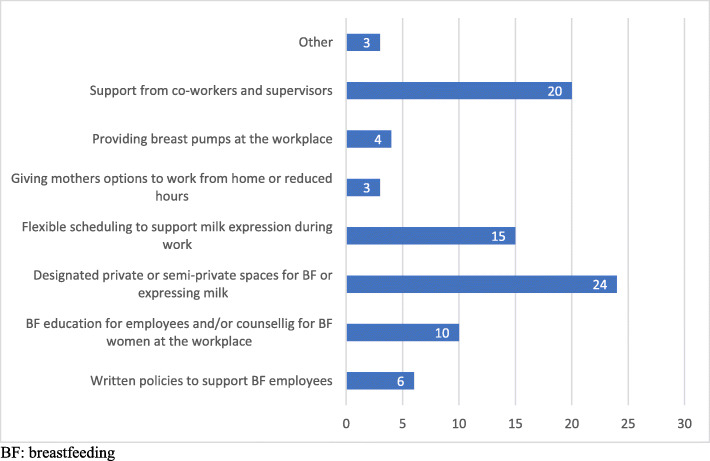
Frequency of workplace interventions to support breastfeeding among working women. BF: breastfeeding

Fig. 2 shows the types and frequency of interventions at the workplace addressed in the literature. Several studies included more than one intervention, hence, were not mutually exclusive. The most common interventions were providing mothers a designated space for breastfeeding or breastmilk extraction (*n* = 24), and the support from co-workers and supervisors (*n* = 20). These were followed by flexible time to express milk (*n* = 15), breastfeeding education or counselling at the workplace (*n* = 10) and institutional written policies to support breastfeeding (*n* = 6). The least common interventions were providing breast pumps (*n* = 4) and giving mothers the flexibility to work from home or reducing in-office hours (*n* = 3).

**Fig. 3:**
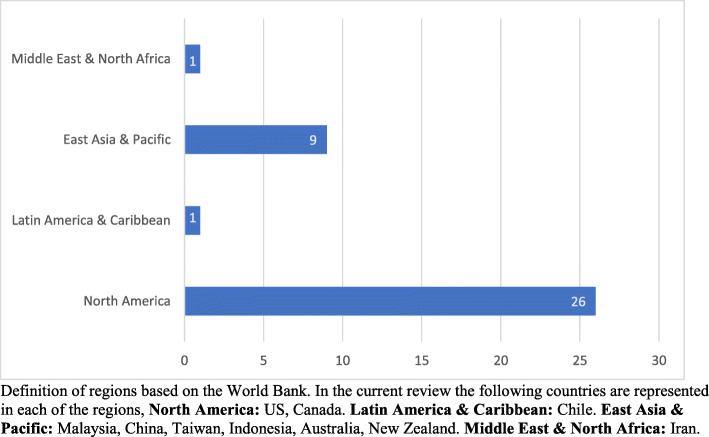
Geographic distribution of studies included in the systematic literature review on workplace interventions to support breastfeeding among working women Definition of regions based on the World Bank. In the current review the following countries are represented in each of the regions, North America: US, Canada. Latin America & Caribbean: Chile. East Asia & Pacific: Malaysia, China, Taiwan, Indonesia, Australia, New Zealand. Middle East & North Africa: Iran

The geographical distribution of the study settings is presented in Fig. 3. The most studies were conducted in North America (70.27%) followed by East Asia and the Pacific (24.32%), Latin America and the Caribbean (2.70%) and the Middle East and North Africa (2.70%). In the rest of the regions – Sub-Saharan Africa, Europe and Central Asia, and South Asia – no eligible studies were reported.

The following section analyses how interventions affected different breastfeeding outcomes among working women including breastfeeding duration (*n* = 17 quantitative and *n* = 1 qualitative); breastfeeding exclusivity (*n* = 4 quantitative and *n* = 1 qualitative); confidence in breastmilk expression (*n* = 4 quantitative); and breastfeeding support from supervisors and co-workers (*n* = 3 quantitative and *n* = 6 qualitative).

### Breastfeeding duration

Seventeen quantitative studies assessed the association between breastfeeding interventions at the workplace and breastfeeding duration including cross-sectional studies (*n* = 13) and prospective cohort and longitudinal designs (*n* = 4) (Table 3). Their quality was graded as moderate (*n* = 6), low (*n* = 7) and very low (*n* = 3). In addition, one qualitative study also addressed breastfeeding duration [34], and its quality was ranked as low (Table 7).

**Table 3:** Workplace breastfeeding interventions and their association with BF^1^ duration, quantitative studies

Author (yr)	Country (City)	Population (n)	Design	Type of BF intervention	Outcome variable	Type of analysis	Effects & associations	Quality assessment
Ahmadi M, Moosavi SM (2013) [17]	Iran (Bandar Abbas)	Employed mothers with healthy children 6–12 months (*n* = 212)	Cross-sectional	-BF education -Designated spaces for BF or expressing milk -Flexible scheduling to support milk expression	Formula use	Bivariate	Infant formula use was significantly lower among women with access to a lactation space compared to those without access (28% vs 59.3%)	Very low
Amin RM, Said ZM, Sutan R, Shah SA, Darus A, Shamsuddin K. (2011) [18]	Malaysia (Petaling district in Selangor)	Working mothers with children 3–12 months who attended health services from governmental health clinics in Petaling (*n* = 290)	Cross-sectional	-Designated spaces for BF or expressing milk	Discontinuation of breastfeeding	Logistic regression	Not having adequate breastfeeding facilities at the workplace was associated with breastfeeding discontinuation (OR^2^ = 1.8, CI 95% 1.1–3.1)	Moderate
Balkam JA, Cadwell K, Fein SB. (2011) [19]	USA	Women participating in the workplace lactation program (3 previous years) & were still employed in the organization (*n* = 128)	Cross-sectional	-BF education -Designated spaces for BF or expressing milk -Provision of breast pumps	Duration of any BF and EBF^3^	Logistic regression	EBF at 6 months: Number of program services received AOR = 1.73 (*p* = 0.003) Any BF at 6 months Return-to-work consultation AOR^4^ = 3.15 (ref: no serv.)	Low
Dagher RK, McGovern PM, Schold JD, Randall XJ. (2016) [20]	USA (Minnesota)	Employed women + 18 years while hospitalized for childbirth (*n* = 817).	Prospective cohort	-Support from colleagues	BF cessation at 6 months postpartum	Cox proportional hazards analysis	No statistically significant results	Moderate
Dozier AM, McKee KS.(2011) [21]	USA (several states)	Infants whose mothers were + 18 years and who had not changed state of residence since birth (*n* = 16,145)	Cross-sectional	-Written policies to support BF employees -Designated spaces for BF or expressing milk -Flexible scheduling to support milk expression	BF duration (< 6 months or > 6 months)	Logistic regression	Not statistically significant results	Low
Fein SB, Mandal B, Roe BE. (2008) [22]	USA	Mothers from the Infant Feeding Practices Study II (IFPSII) who concurrently worked and BF at some point during their infant’s first year of life (*n* = 810)	Prospective cohort	- Flexible scheduling to support milk expression	Duration of BF (weeks)	Regression and censored regression models	Duration of BF after return to work (weeks): Pump only (Marginal Effect −7.11), Pump and feed directly (Marginal Effect − 2.94) Neither pump nor feed during day (Marginal Effect − 11.77) Feed infant directly (ref.)	Low
Jacknowitz A. (2008) [23]	USA	Births of women working (1989–1999) from the National Longitudinal Survey of Youth 1979 and the Children of the National Longitudinal Survey of Youth 1979 (*n* = 1506)	Cross-sectional	-Work from home (i.e., home-office) or reduced hours	BF at 6 months among those who initiated BF	Probit model	BF at 6 months was not significantly associated with the mother’s perception of flexible work schedule availability	Moderate
Kozhimannil KB, Jou J, Gjerdingen DK, McGovern PM (2016) [24]	USA	Women between 18 and 45 years who gave birth in 2011–2012 and were employed at the time of the Listening to Mothers III survey (*n* = 550)	Cross-sectional	-Designated spaces for BF or expressing milk -Flexible scheduling to support milk expression	BF at 1 week and 6 months (any/exclusive) and BF (months)	Logistic regression	Women with sufficient break time were 2.6 times more likely to BF exclusively and 3.0 times more likely to BF at all at 6 months than women without access to break time or private space. Women with access to breaks and space were 2.3 times more likely to BF exclusively at 6 months	Moderate
Mao ZY, Lin XH, Tai XJ, Wang J. (2018) [25]	China (Beijing)	Pregnant women residents in Beijing, who participated in Beijing’s two-child women’s maternity survey and were qualified according to the universal two-child policy and have already given birth to the second child and had complete information (*n* = 247)	Cross-sectional	-Designated spaces for BF or expressing milk	BF duration	Cox Proportional Hazards Regression Model	Women with an independent breastfeeding room in the workplace were less likely to discontinue breastfeeding HR = 0.371	Moderate
Scott VC, Taylor YJ, Basquin C, Venkitsubramanian K. (2019) [26]	USA (North and South Carolina)	Adult female employees (+ 18 y) of the health care system who had been employed for > 6 months, who had BF in the past 3 years (*n* = 165)	Cross-sectional	-Support from colleagues	BF duration (< 6 months, 6–12 months, or > 1 year); EBF; duration of EBF (< 4, 4–5, and 6 months); job satisfaction	Linear and quantile regression models, ordinal probit regressions, binary logistic regressions	BF duration: Not statistically significant results EBF: organizational support AOR = 1.81EBF duration: managerial support: AOR1.47	Low
Smith-Gagen J, Hollen R, Tashiro S, Cook DM, Yang W. (2014) [27]	USA	Data on BF laws enacted by states from the National Council of State Legislatures. Data from NHANES 2003–2010 for BF practices	Cross-sectional	-Written policies to support BF employees -BF education -Designated spaces for BF or expressing milk -Flexible scheduling to support milk expression	Proxy report of infants being BF for at least 6 months	Multivariable logistic regression	Infant feeding at 6 months associated with: -Jury duty exemption for BF mothers (AOR = 1.66), having a private area in the workplace to express breastmilk (AOR = 1.34), having break time to BF or pump (AOR = 1.23); Enforcement pumping (AOR: 2.02)	Moderate
Spatz DL, Kim GS, Froh EB. (2014) [28]	USA (Philadelphia)	Employees who had filed for maternity leave between 2007 and 2011 in the Children’s Hospital of Philadelphia (*n* = 545)	Cross-sectional	-Written policies to support BF employees -BF education -Designated spaces for BF or expressing milk -Flexible scheduling to support milk expression -Provision breast pumps	Duration at 6 and 12 months	Descriptive	Compared to CDC national data, the intervention hospital had a significantly higher BF rate at 6 months (78% at the intervention site vs 42%) and at 12 months (32% vs 25 respectively)	Very low
Spitzmueller C, Wang ZX, Zhang J, Thomas CL, Fisher GG, Matthews RA, et al. (2016) [29]	USA	Subsample of IFPS II of women who returned to paid work within the first year of infant’s life, and women who were BF when they returned to work (*n* = 859)	Longitudinal	-Support from colleagues	BF goals intentions, BF duration after returning work	HLM, Cox proportional hazards regression	Perceptions of workplace BF support were associated to prenatal BF goal intentions (ß = .11) and contributed to predict prenatal BF goal intentions by around 10%. BF goal intentions were negatively associated with the hazard rate of cessation of BF after return to work (**ß** = 0–.16).	Low
Tsai SY. (2013) [30]	Southern Taiwan	Employed mothers at a large electronics manufacturer company in Southern Taiwan (*n* = 715)	Cross-sectional	-Designated spaces for BF or expressing milk -Flexible scheduling to support milk expression -Support from colleagues	Continuing BF after returning to work (in months)	Multiple logistic regression	For mother who continued BF for more than 6 months there was an association with less work hours per day (OR = 2.66), access to an independent lactation room (OR = 2.38), using pumping breaks (OR = 61.6) and encouragement by colleagues to use breaks (OR = 2.44)	Low
Wallenborn JT, Perera RA, Wheeler DC, Lu J, Masho SW. (2019) [31]	USA (Virginia)	Nationally representative data of pregnant women and their children until 1 year of age (Infant Feeding Practices Survey (IFPS) II) (*n* = 1198)	Prospective cohort	-Support from colleagues	BF duration (# of weeks infant was BF) and EBF	Structural equation modelling path analysis	Direct effect of: workplace support on BF confidence (0.63). Similar effects on EBF. Indirect significant effect: workplace support on BF duration through confidence in BF (0.58)	Low
Wambach K, Britt E. (2018) [32]	USA (Mid-western region)	Registered Nurses employed in a large urban teaching hospital system for children (*n* = 78)	Cross-sectional	-Designated spaces for BF or milk expression -Support from colleagues	BF duration	Analysis of variance, Pearson coefficient	Perceived support associated with longer BF duration: Pearson coefficient 0.34	Very Low
Waite WM, Christakis D. (2015) [33]	USA (Seattle)	Female employees of 2 sites – a hospital and a large corporation – who had a child born within the last 5 years (*n* = 531)	Cross-sectional	-Designated spaces for BF or expressing milk -Flexible scheduling to support milk expression -Support from colleagues	BF duration in weeks.	Linear regression models	BF duration was not significantly associated with support score or with any specific domain.	Low

^1^ *BF* Breastfeeding, ^2^ *OR* Odds ratio, ^3^ *EBF* Exclusive breastfeeding, ^4^*AOR* Adjusted odds ratio

Four studies evaluated the association between organizational support from colleagues and managers and breastfeeding duration among working women [20, 26, 29, 31]. Three of the studies reported that support from coworker or managers/supervisors did not have a significant association with duration of breastfeeding. Dagher et al. [20] conducted a survival analysis in Minnesota, United States (US), to estimate the hazard ratio of breastfeeding cessation during the first 6 months after childbirth among women who initiated breastfeeding and returned to work adjusting for different employer factors, among them the support from colleagues and supervisors, which was not statistically significant. Similarly, Scott et al. [26], using data from a cross-sectional survey of employees in a large integrated health care system in North and South Carolina, US, found that while managerial support and organizational support increased job satisfaction and the odds of prolonging exclusive breastfeeding, no significant associations were found between organizational, managerial, and co-worker support and overall breastfeeding duration. Waite and Christakis [33] assessed if support (i.e. score support and its specific domains) was associated with breastfeeding duration among female employees of two sites in Seattle, US, and did not found significant associations in either of the sites.

However, two studies suggest that the effect of workplace support on breastfeeding duration could be indirect. Spitzmueller et al. [29] studied women who returned to work within the first year of life of their infants and were still breastfeeding when returned to work using a subsample from the US Infant Feeding Practice Survey II (IFPS), and found that workplace support was significantly associated with prenatal breastfeeding intentions (HR = 0.11), and, in turn, breastfeeding intentions were negatively associated with the hazard rate of cessation of breastfeeding when returning to work (HR = -0.16). Similarly, Wallenborn et al. [31] also used the IFPS to assess pregnant women and their children until 1 year of age, and found a direct significant effect of workplace support on breastfeeding confidence (ß = 0.63), and an indirect effect of workplace support on breastfeeding duration through confidence in breastfeeding (ß = 0.58).

Duration of breastfeeding was also assessed when combining different strategies to promote breastfeeding at the workplace (*n* = 2). For example, Tsai [30] assessed the association of designated spaces for breastfeeding or breastmilk extraction, flexible time to express milk, and support from co-workers and continuation of breastfeeding after returning to work among employed women at a large manufacturing company in South Taiwan. The author reports increased odds of continued breastfeeding at 6 months with the encouragement from colleagues to use pumping breaks (OR = 2.44). The association was about the same magnitude as other intervention areas such as access to an independent lactation room (OR = 2.44). On the other hand, a cross-sectional study among registered nurses employed in a large urban teaching hospital system in the US [32] assessed the correlations between breastfeeding duration, breastfeeding institutional support, and breaks to extract milk. While breastfeeding institutional support was positively rated, the only significant correlation was between break time and breastfeeding duration (r = .34).

Two studies examined the association between having designated spaces for breastfeeding or breastmilk extraction and breastfeeding discontinuation [18, 25]. In a study of working mothers in Sengalor, Malaysia [18], not having adequate lactation space at the workplace was associated with increased odds of breastfeeding discontinuation (OR = 1.8). Similarly, in a cross-sectional study in Beijing, China, Mao et al. [25] reported that women with an independent breastfeeding room at the workplace were less likely to discontinue breastfeeding (HR = 0.37). A study in Iran [17] documented that infant formula use among working mothers with infants 6–12 months was significantly lower among those with access to a lactation space (28%) than among those without it (59.3%).

When designated spaces for breastfeeding are combined with other interventions there are some mixed findings. For example, in a study using the 2009 National Immunization Survey in the US, Dozier & McKee [21] could not find a significant association between breastfeeding duration at 6 months with type of worksite breastfeeding statute in place, although the sample included all mothers and not just those in or returning to the workforce. Balkam et al. [19], assessed the association of a program in the US that included prenatal class, telephone support from a nurse during maternity leave, return to work consultation with a nurse and access to a lactation room. The authors reported that the return to work consultation was associated with any breastfeeding at 6 months (AOR = 3.15) compared to women without this service, but the rest of the services, including the lactation room, did not yield significant associations. On the other hand, Smith-Gagen et al. [27], in a study from the US using nationally representative cross-sectional data, reported that breastfeeding at 6 months was significantly associated with having a private area in the workplace to express milk (AOR = 1.34), as well as with having break time to feed or express breastmilk (AOR = 1.23). In another cross-sectional study, Kozhimannil et al. [24] found that among working women who participated in the Listening to Mothers III survey, those with sufficient break time to extract milk were 3 times more likely to breastfeed at 6 months than women without break time or private space to extract breastmilk. Similarly, a cohort study from the IFPD II [22] suggest the relevance of having the support and flexible time to extract milk in breastfeeding duration; compared to women who feed their infant directly, those who did not pump or feed during their workday had a significant decrease in breastfeeding duration (marginal effect of − 11.77), compared to those who pumped systematically (marginal effect − 7.11) or those who pumped and fed the baby directly during the day (marginal effect − 2.94). Hence, it highlights the relevance of flexible times to extract or breastfeed the baby during work hours. Spatz et al. [28] reported that in a hospital (Philadelphia, US) with a lactation program for employees, there was a significant larger prevalence of breastfeeding at 6 and 12 months when compared with Center for Diseases Control and Prevention (CDC) national estimates; the program included lactation space, counseling, and pump loans.

Only one study assessed the association between flexibility in working schedule. Based on a national survey from the US, Jacknowitz [23] did not find a significant association between any breastfeeding at 6 months (among those who has started breastfeeding) and the mother’s perception of flexible work schedule availability.

In convergence to the quantitative findings, one qualitative study informed by Baeza et al. [34] conducted among Chilean working mothers enrolled in the public health system, highlighted that mothers identified returning to work as one of the main reasons of early weaning, especially when there are no spaces designated to express breastmilk at the workplace.

In summary, breastfeeding duration among working mothers is indirectly associated with organizational support from co-workers, as their support can promote prenatal breastfeeding intentions and breastfeeding confidence. Having an adequate lactation room or space, protects from breastfeeding discontinuation or introduction of BMS. When combined with flexible time to extract breastmilk or breastfeed, having lactation spaces tends to be positively associated with breastfeeding duration.

### Exclusive breastfeeding

Four cross-sectional studies assessed the association of breastfeeding interventions at the workplace and breastfeeding exclusivity (Table 4). Their quality was graded as low (*n* = 2) and very low (n = 2). Additionally, one qualitative study complemented this body of literature [35], its quality was ranked as low (Table 7).

**Table 4:** Workplace breastfeeding interventions and their association with BF exclusivity, quantitative studies

Author (yr)	Country (City)	Population (***n***)	Design	Type of BF intervention	Outcome variable	Type of analysis	Differences & associations	Quality assessment
Bai Y, Wunderlich SM. (2013) [42]	USA (New Jersey)	Female staff and faculty + 18 years, currently BF or had BF within 18 months prior to the study (*n* = 113)	Cross-sectional	-Designated spaces for BF^1^ or expressing milk -Support from colleagues	Duration of EBF^2^	Pearson’s r correlation analysis	Positive and significant correlation between duration of EBF and workplace support – supervisor support, lactation space (r = 0.26), and technical support – fridge, pump (r = 0.71)	Low
Basrowi RW, Sulistomo AB, Adi NP, Vandenplas Y. (2015) [43]	Indonesia (Jakarta)	Female employees of 3 government offices and 3 factories whose children were between 6 and 36 months old (*n* = 186)	Cross-sectional	-BF education -Designated spaces for BF or expressing milk	EBF at 6 months	Chi-square	EBF at 6 months was higher among women with a dedicated lactation space (OR^3^ = 2.62) and among women with a breastfeeding support program at workplace (OR = 5.93)	Very low
Dabritz HA, Hinton BG, Babb J. (2009) [44]	USA (California)	Mothers who resided in Yolo County at the time of delivery, infant was between 0 and 8 months old at the time they signed up to participate (*n* = 399)	Cross-sectional	-Designated spaces for BF or expressing milk -Flexible scheduling to support milk expression -Support from colleagues	Almost EBF, BF and formula feeding (mixed feeding), infant formula feeding only (at 6 months)	Polytomous logistic regression that incorporated a proportional odds model	Not statistically significant results	Low
Smith JP, McIntyre E, Craig L, Javanparast S, Strazdins L, Mortensen K (2013) [45]	Australia	Female employees with children aged two years and younger (*n* = 356)	Cross-sectional	-Written policies to support BF employees -Flexible scheduling to support milk expression -Designated spaces for BF or expressing milk -Work from home (i.e., home-office) or reduced hours -Support from colleagues	EBF	Bivariate	EBF was significantly more prevalent among women who had flexibility to BF or express milk at the workplace, or worked in a place with a written policy to support breastmilk expression and BF at work	Very low

^1^ *BF* Breastfeeding, ^2^ *EBF* Exclusive breastfeeding, ^3^ *OR* Odds ratio

A study in Indonesia [43], reported that exclusive breastfeeding at 6 months was significantly higher among working women with lactation space (OR = 2.62) and a breastfeeding support program (OR = 5.93) compared to working women without such services. Similarly, Bai and Wunderlich [42] estimated a positive and significant correlation in a study in the US between exclusive breastfeeding and workplace support (r = 0.26) and technical breastfeeding support at the workplace (i.e. access to a fridge, pump) (r = 0.71). Smith et al. [45] documented that exclusive breastfeeding was more prevalent among Australian women employed at workplaces providing flexibility to express or breastfeed, and with written policies supporting breastfeeding. Nevertheless, the only study that adjusted for confounders did not find a significant association between workplace interventions and exclusive breastfeeding; this study was conducted in California, US [44] and assessed the association of having designated spaces to express milk, flexible time to extract breastmilk and organizational support for breastfeeding and exclusive breastfeeding (or almost exclusive breastfeeding) at 6 months.

Only one qualitative paper addressed the outcome of exclusive breastfeeding. Abdulloeva and Eyler [35] conducted a documentary review of written policies and policy statements about lactation support in worksites including time and space to express milk. Then, this was correlated to the states' breastfeeding rates. The findings indicated a positive correlation between having such policies and the exclusive breastfeeding rate at the state level.

Breastfeeding interventions at the workplace can support EBF. However, literature addressing this association is limited in quality, and the only study that adjusted for covariates did not report a significant association between interventions at the workplace and EBF.

### Confidence in breastmilk extraction and breastfeeding at the workplace

Four cross-sectional studies addressed the interplay between breastfeeding interventions at the workplace and the confidence of working women in using lactation spaces, breaks, and pumps to achieve their breastfeeding goals after returning to work (Table 5). Their quality was graded as low (*n* = 2) and very low (*n* = 2).

**Table 5:** Utilization of workplace breastfeeding interventions and its association with breastmilk expression/ pumping behaviors, quantitative studies

Author (yr)	Country (City)	Population (n)	Design	Type of BF intervention	Outcome variable	Type of analysis	Effects & associations	Quality assessment
Henry-Moss D, Abbuhl S, Bellini L, Spatz DL. (2018) [46]	USA (Philadelphia)	Women working at Penn Medicine facility and had pumped milk at work within the previous 5 years (*n* = 151)	Cross-sectional	-Designated spaces for BF^1^ or expressing milk	Breast pumping duration	Bivariate	Women who had pumped for at least one child reported reaching their personal pumping goal, and a significantly longer duration.	Very low
Snyder K, Hansen K, Brown S, Portratz A, White K, Dinkel D. (2018) [47]	USA (Nebraska)	Women returning to work while breastfeeding (*n* = 1002)	Cross-sectional	-Designated spaces for BF or expressing milk -Flexible scheduling to support milk expression - Support from colleagues	Breast pumping duration	Chi-square goodness of fit tests.	Breast pumping duration significantly varied with type of work, as well as other factors such as employer support and meeting BF goals.	Very low
Tsai SY. (2014) [48]	Southern Taiwan	Employed mothers at a large electronics manufacturer company in Southern Taiwan (n = 715)	Cross-sectional	-Designated spaces for BF or expressing milk -Support from colleagues	Use of expression breaks, use of lactation rooms, BF duration	Logistic regression	Significant association between: -partner’s BF support and use of breaks (AOR^2^ = 1.43) and use of lactation room (AOR = 1.66), -partner’s encouragement to use the lactation room and use of breaks (AOR = 6.64); lactation room (AOR = 7.35) -partner’s encouragement to use milk expression breaks and use of breaks (AOR = 3.23); lactation room (AOR = 2.64) -partner’s support intention to keep BF and use of breaks (AOR = 2.63=, lactation room (AOR = 2.10) Partner’s support increased the odds of continuing BF.	Low
Tsai SY. (2014) [49]	Southern Taiwan	Employed mothers at a large electronics manufacturer company in Southern Taiwan (n = 715)	Cross-sectional	-Designated spaces for BF or expressing milk -Support from colleagues	Use of pumping breaks	Logistic regressions	Associations between use of pump breaks and higher education (AOR = 2.33), type of work station (AOR = 1.51), awareness of pumping breaks (AOR = 4.1), having encouragement of colleagues to use the breaks (AOR = 1.76), better awareness of BF benefits (AOR = 1.08), perceptions that taking the breaks can reduce work efficiency (AOR = 0.55)	Low

^1^ *BF* Breastfeeding, ^2^*AOR* Adjusted odds ratio

These studies highlight that confidence in breastmilk expression at the workplace is related to four aspects: individual characteristics of working mothers, type of employment, partners’ support, and support from colleagues and supervisors. At the individual level, a study in a medical facility in Philadelphia, US [46] reported that working women who had prior pumping experience, were more likely to reach their pumping goals and duration when provided with lactation space and breaks. While in a study of working women in a manufacturing company in South Taiwan, Tsai [49] reported that maternal education and awareness of pumping breaks were significantly associated with pumping (AOR = 2.33 and 4.1, respectively). In addition, the type of employment was also associated with the use of pumping breaks (AOR = 1.51). This is consistent with Snyder et al. [47], who found that among women returning to work while breastfeeding, pumping duration and breastfeeding support significantly differed by the type of work they performed. Support from colleagues and supervisors to use breaks seems to be a relevant factor of women’s confidence, which was also supported by Tsai’s study [49]. In addition, the support from partners was also found to be a relevant determinant in women’s confidence in using lactation spaces and pumping breaks [48].

Evidence suggest that women’s confidence in using lactation spaces and pumping breaks is associated with different ecological levels including individual characteristics (e.g. prior experience, education, type of work) and interpersonal factors (e.g. support from colleagues and partners).

### Organizational support and breastfeeding

Three cross-sectional studies assessed attitudes of co-workers and supervisors towards breastfeeding (Table 6). Their quality was assessed as moderate (*n* = 2), low (*n* = 1) and very low (*n* = 1). In addition, six qualitative analyses (*n* = 6) complemented the results related to perceived support (see Table 7). The qualitative studies were based on different approaches such as phenomenological, ethnography and online open-ended questionnaire. The quality of the studies was assessed as moderate (*n* = 1), low (*n* = 2) and very low (*n* = 3).

**Table 6:** Workplace breastfeeding interventions and their association with BF support, quantitative studies

Author (yr)	Country (City)	Population (n)	Design	Type of BF intervention	Outcome variable	Type of analysis	Effects & associations	Quality assessment
Seijts GH, Yip J. (2008) [50]	Canada	Alumni of a large Canadian business school, living in Canada at the time of the study (*n* = 220)	Cross-sectional	-Support from colleagues	Support for BF accommodation in the workplace	Pearson correlation ANOVA	Employees with children reported stronger support for BF accommodations (i.e. lactation space), this was mediated by knowledge	Very low
Suyes K, Abrahams SW, Labbok MH (2008) [51]	USA (Southeastern)	Employees of a corporation that has a reputation of being “family friendly” (*n* = 407)	Cross-sectional	-BF education -Designated spaces for BF or expressing milk -Flexible scheduling to support milk expression -Provision of breast pumps -Support from colleagues	Index of BF Attitudes (IBA)	Linear regression	Having had a co-worker who BF or expressed milk at work was associated with 2.4 point increase in average IBA score. Having ever breastfed was also positively associated with the score.	Low
Zhuang J, Bresnahan M, Zhu Y, Yan XD, Bogdan-Lovis E, Goldbort J, et al. (2018) [52]	USA	Working adults (males and females) drawn form a representative sample of almost every state (*n* = 1000, *n* = 168 women who were BF at the time of the interview)	Cross-sectional	-Designated spaces for BF or expressing milk -Provision of breast pumps -Other	Stigma about BF, intention to help a nursing coworker, self-efficacy of pumping	ANOVA, bivariate correlations. HLM and SEM models	Behavior intention to help BF coworkers was explained by perception of fairness, coworker support and stigmatization (47% of the variance). “Ick response” was positively associated with stigma, and negatively associated with support and fairness. Self-efficacy to pump at work. For women still BF, models showed that perceptions of fairness (**ß** = 0.3) and coworkers’ support (ß = 0.24) were positively associated (relationships were confirmed by the SEM)	Low

**Table 7:** Workplace breastfeeding interventions assed through qualitative designs

Author (yr)	Country (City)	Population (n)	Design	Type of BF intervention	Data Collection	Type of analysis	Findings	Quality assessment
Abdulloeva S, Eyler AA. (2013) [35]	USA	State and university employees (*n* = 50. Policies of all 50 states)	Investigation-action	-Written policies to support BF employees	Documentary review of written policies or policy statements & state breastfeeding rates from breastfeeding report card	Content analysis	−11 states had lactation policies with a detailed description of time and space to express milk. -Significant correlation between State law and 6 months EBF rates.^1^	Low
Anderson J, Kuehl RA, Drury SA, Tschetter L, Schwaegerl M, Hildreth M, Bachman C, Gullickson H, Yoder J, Lamp J. (2015) [36]	USA	Business representatives in a rural Midwestern city (*n* = 32)	Phenomenological	-Support from colleagues	Three focus groups	Thematic analysis	- Interpersonal communication is important to enhance workplace BF support. - Multiple factors like age and the position of the BF employee can affect interpersonal communication about workplace BFsupport.	Low
Baeza WB., Henríquez KF, Prieto GR (2016) [34]	Region on Araucania, Chile	Working mothers using the public health system & had breastfed at least 1 month and were or are in maternity leave (*n* = 65)	Grounded Theory	-BF education -Designated space for BF or expressing milk -Other	Five focus groups, five in-deep interviews and 30 telephonic interviews	Thematic analysis	-Mothers recognize lack of knowledge of BF law, policies and rights, lack of the technique of breastmilk extraction, and storage inside the worksite -Returning to work is one of the reasons mentioned by mothers for early weaning, especially when there are no lactation spaces at the worksite.	Low
Bai YK, Wunderlich SM, Weinstock M. (2012) [37]	USA (New York)	Managers and representatives from human resources at companies with 500 or more employees (n = 20)	Phenomenological	- Support from colleagues -Other	Phone or in-person interviews	Content analysis	-BF support can be helpful to recruit and hold employees. But, the ‘encouragement not to give up breastfeeding early’ was less appreciated as a benefit to the company. -Men, single women or mothers that decided not to breastfeed are unsupportive of a mother-friendly environment. -Space limitation for nursing and pumping rooms, and negative employee dynamics are barriers of workplace BF support. There is also a perception of decreased productivity.	Moderate
Bradford VA, Walkinshaw LP, Steinman L, Otten JJ, Fisher K, Ellings A, O’Leary J, Johnson DB. (2017) [38]	USA (Washington)	Women and men of 110 organizations of four targeted sectors: hospitals, clinics, early care and education settings, and worksites (*n* = 125)	Investigation-action	- Written policies to support BF employees - Support from colleagues	Semi-structured interviews	Thematic analysis	-Federal and states laws, policies and performance bring awareness and motivates action. -Organizations have limited financial resources for the development and implementation of supportive breastfeeding policies. -Organization structure affects the ability to develop and implement supportive breastfeeding policies. -Positive experiences facilitate supportive policies and practices.	Moderate
Froh EB, Spatz DL (2016) [12]	USA (Philadelphia)	Female employees of The Children’s Hospital of Philadelphia (*n* = 410)	No design (two open questions were added to a survey of a quantitative study - cohort model)	-Written policies to support BF employees - BF education - Support from colleagues	A quantitative survey was deployed to all women (1362), the survey included two qualitative questions. It was optional to answer them.	Content analysis	-Supporting BF mothers after returning to work is key to enable them meeting their BF goals, but even when an institution has a strong lactation policy, mothers may be unaware of the policy, feel internal pressure to avoid frequent pumping breaks, and possibly feel unsupported by both peers and supervisors.	Very low
Majee W, Jefferson UT, Goodman LR, Olsberg JE. (2016) [39]	USA (Missouri)	Low-income employed and unemployed breastfeeding mothers and key employers of rural area (n = 10 / 7)	Phenomenological	-BF education -Flexible scheduling to support milk expression - Support from colleagues	One focus group and individual interviews and document review	Content analysis	- Workplace milk expression must shift from a reactive stance to regular education for mothers and employees, to allow creating a supportive environment enabling mothers’ BF goals.	Low
Payne D, James L (2008) [40]	New Zealand	Working mothers of different ethnicities (*n* = 34)	Phenomenological	- Designated space for BF or expressing milk -Flexible scheduling to support milk expression - Support from colleagues	Semi-structured, open-ended, in-depth and interactive interviews	Thematic analysis	-Implementation of a BF intervention depends on superior’s and colleague’s attitudes towards breastfeeding. Males were less supportive. -Lack of access to a lactation space and limited time to pump influenced women’s decision either not to return to paid employment or to discontinue breastfeeding	Very low
Spagnoletti BRM, Bennett LR, Kermode M, Wilopo SA, Spagnoletti B (2017) [41]	Indonesia (Yogykarta)	Working women who had given birth in the last 2 years (n = 20)	Ethnography	- Designated space for BF or expressing milk -Flexible scheduling to support milk expression -Work from home (i.e., home-office) or reduced hours	In-depth interviews, focus groups, semi-structured interviews and participant observation	Descriptive analysis	-Majority of women did not have access to a space designated for breastfeeding. Among those who had it, feeling comfortable was imperative to their ability to express breastmilk.	Very low

^1^ Study with mixed methods, explored correlation between status of legislation at State level and Exclusive breastfeeding (EBF) rate

Two of the cross-sectional studies [50, 51] highlighted that employees and co-workers with children and experience in breastfeeding or milk expression at the workplace reported stronger support for breastfeeding accommodations. Seijts and Yip [50] suggest this can be mediated by an increased level of knowledge about the benefits of breastfeeding. In a study conducted in the US by Zhunag et al. [52], hierarchical linear models and structural equations suggest that behavioral intentions to help breastfeeding co-workers was mainly explained by perceptions of fairness and support, and stigmatization (explaining 47% of the variance). They analyzed the “ick response” caused by human milk and found it to be positively associated with stigmatization and negatively associated with perceptions of fairness and support. The authors further documented that women’s self-efficacy in expressing breastmilk at work was significantly associated with fairness (ß = 0.3) and support (ß = 0.24).

The qualitative studies confirmed that breastfeeding interventions among working women are fundamental to fulfill their goals, but that they need to be accompanied with an actual sense of support, as women can feel discouraged from pumping due to peer pressure [12], and lack of practical support from peers and supervisors [12, 40]. From a managerial perspective, breastfeeding support can be helpful to recruit and hold employees, but, in addition, the actual value of breastfeeding needs to be understood by co-workers and managers, as it is common to encounter barriers such as negative perceptions about breastfeeding support, and the perception that it reduces productivity [37]. In a phenomenological study in the US, Anderson et al. [36] identified important interpersonal aspects about successful breastfeeding at the workplace, which can be affected by age and the position of the employee. Spagnoletti et al. [41] further highlight, through their ethnographic study in Indonesia, that for women to pump milk, they need to have a lactation space, but, in addition, they need to feel comfortable in doing so. As reported by Majee et al. [39] in addition to transforming pumping as a default option in the work space, it is necessary to create a supportive working environment based on education for the mothers, as well as employees in general.

Studies suggest that support for breastfeeding mothers at the workplace is mediated by experience and knowledge from co-workers. In addition, perceptions of fairness and stigmatization of breastfeeding in work/professional settings are important predictors of lack of support. Qualitative studies describe that support and feeling comfortable in breastfeeding or extracting breastmilk at the workplace is fundamental in achieving successful breastfeeding interventions at the workplace. Negative perceptions about the impact of breastfeeding on productivity also impact organizational effective support.

## Discussion

Women in reproductive ages have an increasing role in the labor market. This is something positive from a gender and economic development perspective. Yet, working mothers need to have equitable conditions to breastfeed due to the important benefits that it confers to mothers and babies in the short and long-term [3, 53]. While a maternity leave is fundamental to promote, protect and support breastfeeding initiation, duration and exclusivity, the reality is that only half (53%) of the countries around the world comply with the ILO standard of at least 14 weeks of leave [54]. Hence, a complementary measure to support breastfeeding among working mothers is through interventions at the workplace, such as lactation rooms, flexible times to express milk, and options to work from home, amongst others.

The findings from our systematic review suggest that there are important differences in the geographic distribution of the interventions assessed, mainly located in North America and East Asia and the Pacific. While this might be a publishing bias, it could also respond to differences in approaches to supporting working mothers, as in several European countries they provide maternity leaves beyond the14 weeks minimum recommended by the ILO [54].

The most frequent strategies and actions implemented to promote, protect and support breastfeeding in the workplace were the provision of a designated private space for breastfeeding or expressing milk (i.e. lactation rooms) and having the support of supervisors or coworkers, followed by allowing flexible scheduling to support milk expression during work and having written policies to support breastfeeding mothers. The least frequent intervention was giving mothers options for returning to work, such as teleworking or working part-time. Some studies considered the combination of different interventions, which is likely to lead to more comprehensive lactation support programs at the workplace including: physical resources (i.e. designated private space with pumping equipment and a cooler or refrigerator for storing milk), organizational resources (i.e. flexible breaks, work arrangement options, on-site-child care), education resources (i.e. prenatal classes, postpartum lactation counseling), and workplace support by establishing a lactation support policy and encourage support from managers and co-workers [55]. According to the findings from the literature review, this comprehensive approach is still uncommon.

The systematic literature review revealed that the quality of studies can be improved. Common problems were biased samples, which compromised internal and external validity. There is a need for studies assessing impacts and effects of interventions to promote, protect and support breastfeeding at the workplace on infant feeding practices, mothers’ self-esteem and work centers’ related outcomes such productivity and abstentionism. Hence, experimental or quasi experimental studies are required to scale workplace evidence-based interventions.

Despite these methodological limitations, the literature suggests that the workplace is an important space to intervene. From an ecological perspective, the workplace is an organizational level in which institutional support for breastfeeding mothers can be fostered. Such support was found to interact with individual level factors linked to breastfeeding intentions and self-efficacy. Having organizations that support in promoting lactation rooms and flexible time to extract breastmilk or breastfeed, is associated with longer breastfeeding duration. Interpersonal factors are also fundamental, including the support from co-workers and partners. Knowledge among co-workers seems to be a way to promote effective organizational support and reducing stigma about its effects on productivity. This suggests that interventions are needed towards guiding firms on how to intervene at the three ecological layers – individual, interpersonal, and organizational – in order to provide equitable work environments for breastfeeding women, and fair nutritional opportunities for their infants.

Workplace can be a relevant space to promote, protect and support breastfeeding among working women. It represents an opportunity to foster gender equity, and the health and nutrition of mothers and infants [56]. But interventions and their impact pathways need to be better understood and documented. It is of paramount importance to conduct implementation science-based studies identifying the scope, effectiveness, adoption, implementation, and maintenance of interventions focused on promoting a breastfeeding-friendly work environment.

Some pending issues that should be addressed in future studies are the adaptations of workplace breastfeeding interventions for firms of different types and size, as well as to address the issue of interventions for women working in the informal economy, who are generally at a greater risk to lack maternity leave coverage [57, 58]. Additionally, it is necessary to address the policy design of breastfeeding interventions at the workplace, namely, if they should be implemented based on regulatory (i.e. laws or enforceable rules), market (i.e. deductions, subsidies) or voluntary (i.e. certifications) policy instruments.

This study had some limitations. First, we did not include grey literature and governmental reports, although it is likely that these would have been more descriptive than evaluative. And second, the review did not include aspects about mothers’ job satisfaction, as it would have added an additional outcome, but we acknowledge this is a relevant aspect when considering breastfeeding interventions at the workplace. Despite these limitations, the current review adds important evidence about the need to conduct studies with more robust methodological designs (i.e. experimental, quasi-experimental, economic evaluations) and in describing the different ecological levels that need to be connected in designing effective interventions for breastfeeding working women.

## Conclusions

Employment should not be a source of inequity for breastfeeding women. Explicit interventions and policies are needed to support working mothers. Ideally all women should have a maternity leave benefit, and this should be complemented by breastfeeding friendly working environments. Despite the challenges unveiled by the quality assessment, it is feasible to underline that for workplace environments to be supportive of breastfeeding, women need to know their rights and be trained about instrumental aspects of breastfeeding, such as extraction and storage of breastmilk. In addition, they need to have adequate physical spaces to breastfeed or extract breastmilk, store it and have the support of managers and co-workers. Organizational support requires written polices, as well as breastfeeding education for mothers, managers and co-workers, as this would increase the chances of a supportive environment promoting efficacy of breastfeeding mothers (i.e. using lactation spaces and pumping breaks) and facilitating a breastfeeding friendly environment at the work place in which women feel confident and without fear of being stigmatized or discriminated. Such integral interventions have seldom been documented in the literature, thus, it is strongly recommended to conduct implementation research and impact evaluations following stronger methodological designs than those reported in the available literature.

## Data Availability

Data analyzed is included in the article.

## Abbreviations

WHO: World Health Organization
UNICEF: United Nations International Children’s Emergency Fund
PRISMA: Preferred Reporting Items for Systematic Reviews and Meta-Analysis
ILO: International Labour Organization
NICE: National Institute for Health and Care Excellence
US: United States of America
IFPS: Infant Feeding Practice Survey
CDC: Center for Diseases Control and Prevention
OR: Odds ratios
AOR: Adjusted odds ratios
